# Quadratic Spline Fitting for Robust Measurement of Thoracic Kyphosis Using Key Vertebral Landmarks

**DOI:** 10.3390/diagnostics15212703

**Published:** 2025-10-25

**Authors:** Nikola Kirilov, Elena Bischoff

**Affiliations:** 1 Department of Orthopedics and Traumatology, Medical University-Pleven, 5800 Pleven, Bulgaria; 2 Faculty of Medicine, Burgas State University “Prof. Dr. Assen Zlatarov”, 8000 Burgas, Bulgaria

**Keywords:** kyphosis, medical image analysis, spine curvature, spline interpolation, thoracic spine

## Abstract

**Objective:** The purpose of this study is to present a kyphosis measurement method based on quadratic spline fitting through three key vertebral landmarks: T12, T8 and T4. This approach aims to capture thoracic spine curvature more continuously and accurately than traditional methods such as the Cobb angle and circle fitting. **Methods:** A dataset of 560 lateral thoracic spine radiographs was retrospectively analyzed, including cases of postural kyphosis, Scheuermann’s disease, osteoporosis-induced kyphosis and ankylosing spondylitis. Two trained raters independently performed three repeated landmark annotations per image. The kyphosis angle was computed using two methods: (1) a quadratic spline fitted through the three landmarks, with the angle derived from tangent vectors at T12 and T4; and (2) a least-squares circle fit with the angle subtended between T12 and T4. Agreement with reference Cobb angles was evaluated using Pearson correlation, MAE, RMSE, ROC analysis and Bland–Altman plots. Reliability was assessed using intraclass correlation coefficients (ICC). **Results:** Both methods showed excellent intra- and inter-rater reliability (ICC ≥ 0.967). The spline method achieved lower MAE (5.81°), lower RMSE (8.94°) and smaller bias compared to the circle method. Both methods showed strong correlation with Cobb angles (r ≥ 0.851) and excellent classification performance (AUC > 0.950). **Conclusions:** Spline-based kyphosis measurement is accurate, reliable and particularly robust in cases with severe spinal deformity. Significance: This method supports automated, reproducible kyphosis assessment and may enhance clinical evaluation of spinal curvature using artificial intelligence-driven image analysis.

## 1. Introduction

Kyphosis is a sagittal plane deformity characterized by an excessive forward curvature of the thoracic spine. It is commonly encountered in various clinical conditions including Scheuermann’s disease, ankylosing spondylitis and osteoporosis, each of which influences the spinal morphology and biomechanics in different ways [1,2,3]. Scheuermann’s disease, a juvenile osteochondrosis, typically presents with anterior vertebral wedging leading to a rigid and pronounced thoracic kyphosis [4]. Ankylosing spondylitis, a chronic inflammatory disease, causes fusion of spinal segments and loss of normal spinal flexibility, which often results in a fixed kyphotic deformation [5]. Osteoporosis, particularly in elderly populations, predisposes patients to vertebral compression fractures, predominantly wedge fractures, which significantly exacerbate kyphotic deformity and impair sagittal balance [6].

Accurate quantification of kyphosis is essential for diagnosis, prognosis, treatment planning and long-term monitoring. The Cobb angle remains the most widely used clinical measure, calculated by drawing intersecting lines on the superior endplate of the uppermost vertebra and the inferior endplate of the lowermost vertebra within the kyphotic segment [7]. Despite its extensive clinical acceptance, the Cobb method has well-documented limitations. It relies on the identification of vertebral endplates which can be ambiguous in cases of vertebral wedging or deformity, common in conditions like Scheuermann’s disease and osteoporotic fractures [8]. Furthermore, the Cobb angle represents a single planar angle between two discrete vertebrae and thus fails to capture the continuous curvature and complex three-dimensional morphology of the spine.

In addition to the Cobb angle, circle fitting methods have been proposed as alternatives to describe spinal curvature by fitting a circle arc to a set of vertebral points or centroids [9]. Circle fitting can provide curvature radius and arc length measurements that better reflect the smooth nature of spinal curvature than linear angles. However, this method also exhibits limitations, particularly in cases of severe or non-uniform kyphotic deformities. Large kyphosis angles and wedge-shaped vertebrae can cause the spinal curve to deviate substantially from a perfect circular arc, resulting in poor fits and inaccurate curvature estimates [10]. The inability of circle fitting to model variations in curvature along the spine limits its utility in clinical scenarios involving complex deformities.

To overcome these limitations, spline interpolation methods have gained attention for spinal sagittal profile assessment. Splines provide a flexible mathematical tool to fit smooth, piecewise polynomial curves through a series of anatomical landmarks. Quadratic and cubic splines can accurately model the gradual change in spinal curvature using relatively few points, allowing the extraction of tangent vectors and curvature values at any point along the spine [11]. These techniques offer the advantage of capturing local curvature changes caused by vertebral wedging or deformity, providing a more detailed and physiologically relevant representation of spinal alignment.

In this study we propose a kyphosis measurement method based on quadratic spline fitting through three key vertebrae: T12, T8 and T4. By calculating the angle between tangent vectors of the spline at T12 and T4, the method quantifies the continuous curvature of the thoracic spine in a manner that inherently incorporates vertebral wedging and regional curvature variations. Compared to the Cobb angle and circle fitting, this spline-based approach offers a potentially more robust and sensitive measure of kyphosis, particularly in cases of severe deformity or complex vertebral anatomy.

## 2. Materials and Methods

This retrospective study was conducted using a dataset of 560 anonymized lateral thoracic spine radiographs obtained from clinical imaging archives. The dataset comprised 210 cases of postural kyphosis, 140 cases of Scheuermann’s disease, 120 cases of osteoporosis-induced kyphosis and 90 cases of ankylosing spondylitis. This ensured a heterogeneous sample with varying anatomical and pathological curvature patterns.

Each image had a reference Cobb angle measurement available, determined using standard clinical procedures. Of the 560 cases, 160 were classified as hyperkyphotic (Cobb angle > 40°), while the remaining 400 were considered within the normal range (≤40°).

Two trained raters (Operator A and Operator B) independently annotated each of the 560 images. To assess intra-rater reliability, each operator performed three repeated annotations per image, spaced over time to minimize memory effects. This resulted in a total of 3360 annotation sessions (560 images × 2 raters × 3 repeats).

During each session, the rater manually selected three anatomical landmarks on the X-ray: the anterior vertebral wall midpoints of T12, T8 and T4. These vertebral levels were chosen because they represent the distal, middle and proximal regions of the thoracic curvature and are easily identifiable on lateral radiographs, thereby reducing interobserver variability [12,13]. These points define the curvature of the thoracic spine and were chosen to approximate the kyphotic apex and boundary levels. Given that visualization of the upper thoracic vertebrae (especially T4) can be partially obscured by shoulder or scapular overlap, a standardized vertebral identification protocol was applied. Vertebral levels were numbered by counting ribs inferiorly from T1 and confirmed using the characteristic morphology of T12 and L1 as reference points. When T4 could not be confidently localized, T5 was used instead and this substitution was documented. Images with substantial rotation, beam angulation or obscured vertebral endplates were excluded from analysis.

To evaluate the robustness of the method to landmark selection and visibility constraints, a sensitivity analysis was conducted on a representative subset of 80 radiographs, spanning the full kyphosis spectrum. In addition to the standard T12–T8–T4 configuration, alternative landmark triplets (T10–T7–T3 and T12–T9–T5) were annotated by a single operator following the same procedure.

**Figure 1:**
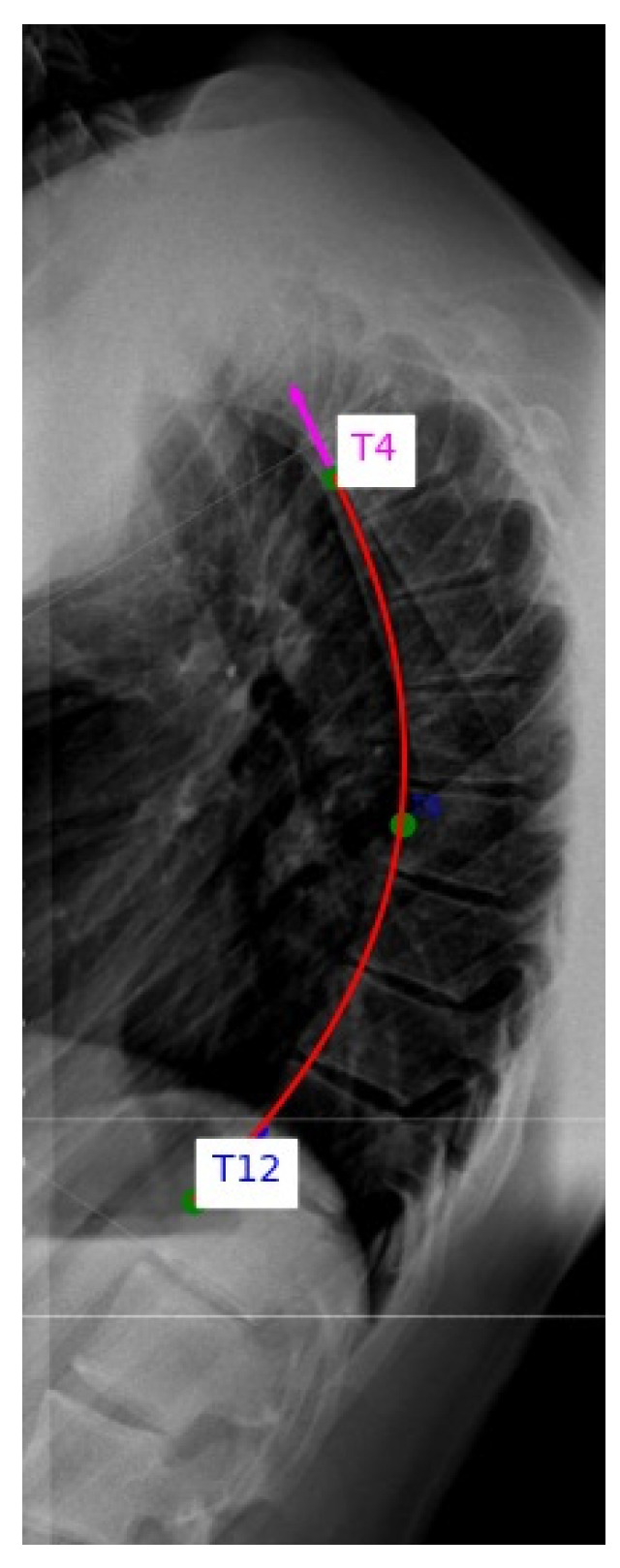
Custom Python tool for thoracic spine curvature analysis. The rater manually selects anterior wall midpoints of the triplet on lateral X-rays. The tool enables interactive landmark selection, automated spline and circle fitting and kyphotic angle calculation.

Annotations, angle calculations and data management were performed using a custom Python 3.13.6 program developed for this study. This program enabled interactive landmark selection, automatic spline and circle fitting, angle computation and systematic recording of all results for downstream analysis (Figure 1).

The first method for estimating thoracic kyphosis involved fitting a second-order B-spline curve through the annotated points using scipy.interpolate.splprep. The curvature angle was then calculated from the angle between the tangent vectors at the endpoints of the fitted spline. The angle *θ_spline_* between the two tangents $\underset{t_{1}}{\to }$ and $\underset{t_{2}}{\to }$ was computed as shown in Equation (1).

(1) $$\theta_{spline}=\cos^{-1}\left(\frac{\underset{t_{1}}{\to }\cdot \underset{t_{2}}{\to }}{\left\|\underset{t_{1}}{\to }\right\|\cdot \left\|\underset{t_{2}}{\to }\right\|}\right)$$

The second method fit a circle to the three landmarks using a least-squares approach.

The circle parameters were estimated by solving the system, shown in Equation (2), given the coordinates of the annotated points (*x_i_*,*y_i_*).

(2) $$\begin{array}{cc} \left[\begin{array}{ccc} 2x_{1} & 2y_{1} & 1\\ 2x_{2} & 2y_{2} & 1\\ 2x_{3} & 2y_{3} & 1 \end{array}\right] & \left[\begin{array}{c} x_{c}\\ y_{c}\\ c \end{array}\begin{array}{c} x_{c}\\ y_{c}\\ c \end{array}\right]=\left[\begin{array}{cc} x_{1}^{2} & y_{1}^{2}\\ x_{2}^{2} & y_{2}^{2}\\ x_{3}^{2} & y_{3}^{2} \end{array}\right] \end{array}$$

Solving for *x_c_*, *y_c_* the radius was calculated as shown in Equation (3).

(3) $$\;r=\sqrt{c+x_{c}^{2}+y_{c}^{2}}\;$$

The angle subtended by the arc between the endpoints was computed using the arctangent difference from the circle center Equation (4):

(4) $$\theta_{circle}=\left|\tan^{-1}\left(\frac{y_{1}-y_{c}}{x_{1}-x_{c}}\right)-\tan^{-1}\left(\frac{y_{2}-y_{c}}{x_{2}-x_{c}}\right)\right|$$

The resulting angle (in degrees) for both methods was recorded and was classified as indicating hyperkyphosis if *θ* > 40°.

To assess how kyphosis severity influenced the accuracy of the two measurement methods, we stratified the dataset into high kyphosis cases (Cobb angle > 60°) and low-to-moderate kyphosis cases (Cobb angle ≤ 60°). Within each group, we compared the mean absolute error (MAE) of the spline and circle methods relative to the gold-standard Cobb angles. This analysis was used to evaluate whether measurement performance degraded in the presence of extreme curvature and whether one method was more robust in such scenarios.

All statistical analyses were performed using Python with relevant packages for reproducibility and transparency. The analysis aimed to evaluate measurement reliability, method agreement with clinical standards and accuracy according to kyphosis severity.

Intra-rater reliability was assessed using the intraclass correlation coefficient (ICC) of type ICC(2,1), which estimates consistency of repeated measurements by the same rater. ICC values were computed separately for each rater (A and B) and each measurement method (spline and circle), based on all three repeats per image.

Inter-rater reliability was quantified using ICC(2,1) across both raters. For this analysis, repeat measurements were first averaged per image and method, then compared across raters to evaluate between-operator agreement.

ICC values range from 0 to 1, with values above 0.75 generally considered good and values above 0.90 indicating excellent reliability.

To assess how closely the automated methods agreed with clinical gold-standard Cobb angle measurements, the following statistical comparisons were performed for each method:

- Pearson correlation coefficient (*r*) with corresponding *p*-values to measure linear association with Cobb angles.
- MAE, calculated as the average absolute difference between method-predicted angles and the corresponding Cobb angle.
- Root Mean Squared Error (RMSE) to evaluate overall magnitude of error, penalizing larger deviations more heavily.
- Paired *t*-tests comparing the mean difference between Cobb angles and the spline or circle method angles to test for systematic bias in the means.
- Levene’s tests for equality of variances to assess whether the measurement variance differed significantly from that of the Cobb angles.
- Receiver Operating Characteristic (ROC) analysis to evaluate binary classification performance for detecting hyperkyphosis, defined as Cobb angle > 40°. The Area Under the Curve (AUC) was computed for both methods.
- Bland–Altman analysis, including the calculation of mean bias (average difference from Cobb angles) and limits of Agreement (LoA), defined as the mean difference ± 1.96 × standard deviation of the differences, which describe the expected range of measurement deviations.
- For the landmark sensitivity analysis, kyphotic angles derived from the alternative landmark triplets were compared to those obtained using the original T12–T8–T4 configuration, which served as the reference. For the analysis subset of 80 images, we ensured an equal distribution of cases with high kyphosis (40 images) and low-to-moderate kyphosis (40 images). Differences in MAE, mean bias and ICC were calculated to assess the impact of varying landmark selection on measurement accuracy and reliability. Paired *t*-tests were performed to evaluate whether the observed differences in MAE between each alternative triplet and the standard configuration were statistically significant. This supplementary analysis was designed to determine whether shifting the presumed kyphotic apex or including adjacent vertebral levels significantly affected curvature estimation.

## 3. Results

Table 1 summarizes the ICC(2) values obtained for both the spline and circle measurement methods across the two raters A and B. For the spline method, rater A achieved an ICC of 0.968, while rater B demonstrated a similarly high ICC of 0.967, indicating excellent repeatability of measurements within each rater. The circle method showed even slightly higher intra-rater reliability with ICC values of 0.973 and 0.970 for raters A and B, respectively.

These results confirm that both measurement methods yield highly consistent angle measurements when repeated by the same rater, with the circle method demonstrating marginally superior intra-rater reliability.

As shown in Table 2, both methods demonstrated excellent inter-rater reliability, with ICC values close to 1. Specifically, the spline method yielded an ICC of 0.979, while the circle method showed a slightly higher ICC of 0.982. These high ICC values indicate a strong agreement between raters regardless of the measurement method used.

This suggests that both the spline and circle methods produce highly reproducible measurements across different raters, supporting their robustness and reliability in clinical or research settings.

Both methods showed strong positive correlations with Cobb angles with Pearson correlation coefficients of 0.851 for the spline method and 0.855 for the circle method (both *p* < 0.001), indicating high linear association between measurements.

In terms of absolute error the spline method achieved a lower MAE of 5.81° compared to 6.80° for the circle method. Similarly, RMSE was lower for the spline method (8.94°) than the circle method (9.63°), indicating slightly better overall accuracy. These correlation and error metrics are summarized in Table 3.

ROC analysis for detecting Cobb angles greater than 40° showed excellent discriminatory performance for both methods, with the circle fitting method achieving an AUC of 0.952 (95% CI: 0.925–0.979) and the spline fitting method achieving a higher AUC of 0.964 (95% CI: 0.937–0.985).

Paired *t*-tests comparing Cobb angles with each method’s measurements yielded non-significant differences (*p* = 0.44 for spline; *p* = 0.06 for circle), indicating no systematic bias in measurement means. Levene’s test also confirmed equal variances between the two methods and the Cobb angles (*p* = 0.78 for spline; *p* = 0.48 for circle).

Bland–Altman analysis revealed a mean bias of −0.93° for the spline method and 2.46° for the circle method, with limits of agreement spanning approximately −16.6° to 18.5° for spline and −16.0° to 20.9° for circle. These additional metrics, including ROC AUC, Levene’s test, Bland–Altman mean bias and limits of agreement are detailed in Table 4.

**Figure 2:**
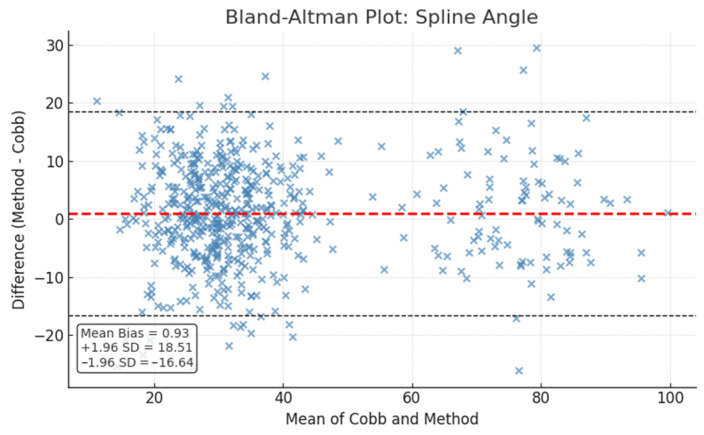
Bland–Altman plot comparing spline method with Cobb angles. The red dashed line represents the mean difference (bias), while the black dashed lines indicate the upper and lower limits of agreement (mean ± 1.96 SD).

**Figure 3:**
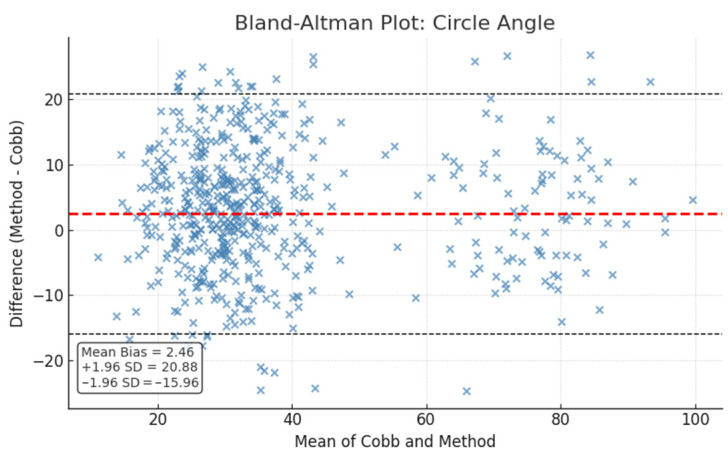
Bland–Altman plot comparing circle method with Cobb angles. The red dashed line represents the mean difference (bias), while the black dashed lines indicate the upper and lower limits of agreement (mean ± 1.96 SD).

**Figure 4:**
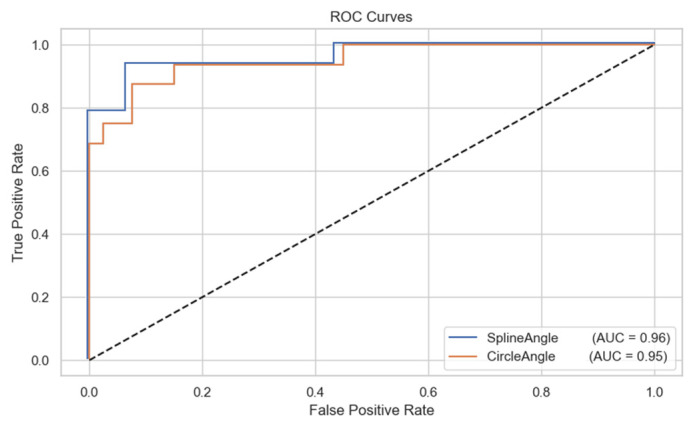
ROC curves for spline and circle methods in detecting Cobb angle > 40°.

Bland–Altman plots (Figure 2 and Figure 3) illustrate the limits of agreement visually, while ROC curves (Figure 4) depict the classification performance of both measurement methods.

We further investigated whether the accuracy of angle measurements differed based on the severity of kyphosis by comparing MAE between cases with high kyphosis angles (>60°) and those with low kyphosis angles (≤60°).

The spline method demonstrated a substantially higher MAE in the high kyphosis group with a mean error of 20.67°, compared to a much lower MAE of 4.97° in the low kyphosis group. Similarly, the circle method exhibited an even greater disparity with a mean MAE of 26.77° for high kyphosis cases versus 5.67° for low kyphosis cases.

These findings suggest that measurement error increases with greater kyphosis severity for both methods with the spline method generally achieving lower errors than the circle method across severity groups. This highlights potential challenges in accurately measuring angles in more severe kyphotic deformities.

The sensitivity analysis assessed how variations in landmark triplets affected kyphosis angle measurements using both spline and circle fitting methods. Using the standard T12–T8–T4 triplet as the reference, alternative configurations (T10–T7–T3 and T12–T9–T5) showed slightly increased MAE and bias for both methods. However, these differences were not statistically significant (paired *t*-test *p* > 0.3 for all comparisons). ICC remained consistently high (≥0.95) across all landmark sets, demonstrating reliable measurement reproducibility regardless of landmark selection. Overall, these results indicate that while landmark choice has a modest impact on measurement accuracy, both spline and circle methods provide robust kyphosis angle estimation across the vertebral triplets (Table 5).

## 4. Discussion

This study presents a quadratic spline-based method for quantifying thoracic kyphosis using three key vertebral landmarks and compares its performance to a traditional circle-fitting approach. Our findings confirm the known advantages of spline-based geometry, including the ability to model continuous spinal curvature more physiologically than circular approximations. While the use of splines is not novel per se, applying them to kyphosis measurement with three easily identifiable landmarks, combined with systematic comparison to circle-fitting and Cobb angles, represents a meaningful methodological refinement with potential clinical relevance. Subtle yet meaningful differences in accuracy and robustness were observed, particularly in cases with severe kyphotic deformity.

The intra-rater reliability values (ICC ~0.97 for both methods) are consistent with prior studies on manual and semi-automated spine angle measurements, which typically report ICC values ranging from 0.90 to 0.98 [14]. The slightly higher ICC for the circle method may reflect the geometric simplicity of fitting a circle to three points, which reduces subjective variability in tangent estimation compared to spline tangents. However, ICC reflects overall reproducibility and does not isolate landmark-dependent variability, which remains operator-dependent in the current study. Future work should incorporate automated landmark detection or sensitivity analyses to quantify and reduce this source of variability. Additionally, formal power calculations for detecting small differences between methods were not performed.

Inter-rater reliability was similarly excellent for both methods (ICC > 0.97), which aligns with previous reports that well-trained raters can reliably identify vertebral landmarks and measure kyphosis angles [15]. This strong agreement supports the clinical utility of both methods in multi-rater or research contexts.

Regarding agreement with Cobb angles the spline method demonstrated marginally superior accuracy as indicated by lower MAE and RMSE, despite nearly identical Pearson correlations (~0.85) and ROC AUC (~0.95) values for both methods. While these differences are statistically small, they may have practical significance in severe deformities or surgical planning, where even minor deviations can affect treatment decisions. The study does not yet provide evidence on how spline-derived measurements would alter diagnosis, treatment thresholds or patient outcomes. Future studies should examine correlations between spline-based metrics and clinical endpoints, including progression risk and patient-reported outcomes. Nevertheless, by modeling the continuous curvature of the spine, the spline approach can better represent subtle vertebral wedging and regional shape variations that are often oversimplified by circular arcs, consistent with Bernstein et al. [16], who highlighted the advantages of spline-based curvature metrics for improving sensitivity in deformity assessment. It is important to note, however, that Cobb angles themselves have limitations, including reliance on vertebral endplate identification and inability to fully capture continuous spinal curvature, which may influence observed measurement agreement.

The Bland–Altman analyses revealed a smaller mean bias and slightly narrower limits of agreement for the spline method, suggesting less systematic deviation from Cobb angles. This supports the idea that quadratic splines provide a more physiologically meaningful representation of spinal curvature than circle fitting, which may oversimplify complex deformities [17]. Both methods’ errors increased substantially in cases with high kyphosis (Cobb angle > 60°) with some deviations exceeding 20°, highlighting the challenges of accurately measuring extreme deformities. Our landmark sensitivity analysis further demonstrated that while altering vertebral landmark selections moderately affects measurement accuracy, it does not fully resolve the challenges posed by shifted kyphotic apex locations or multi-level deformities. This trend aligns with prior reports, where vertebral rotation, wedging and altered anatomy complicate landmark placement and geometric assumptions [8,18,19]. Notably, the spline method maintained lower errors than the circle method in this subgroup, suggesting greater robustness. However, these large deviations underscore the need for future validation using 3D imaging or additional vertebral landmarks, as accurate quantification in severe kyphosis is critical for treatment planning, surgical decision-making and longitudinal monitoring.

From a biomechanical perspective, accurate characterization of thoracic curvature is essential for understanding spinal load distribution, sagittal balance and surgical planning. Quadratic spline-based methods provide a more physiologically realistic representation of continuous spinal curvature compared to circular approximations, which may enhance biomechanical modeling, improve assessment of spinal stress and better predict deformity progression.

Limitations of this study include its retrospective design, the limited number of severe kyphosis cases and the absence of validation against more accurate three-dimensional reference standards (e.g., 3D CT reconstruction). Using only three vertebral landmarks provides a simplified representation of thoracic curvature and may not capture intermediate deviations in multilevel or complex deformities. Future implementations should consider incorporating additional vertebral points. Additionally, etiology-specific subgroup analyses were not performed due to sample size constraints, which limits assessment of differential accuracy. Further studies should stratify by etiology to evaluate potential clinical differences and validate these findings in larger, prospective cohorts, exploring integration with automated landmark detection algorithms to reduce operator dependency.

## 5. Conclusions

This study demonstrates that quadratic spline fitting provides a reliable, accurate and physiologically meaningful method for measuring thoracic kyphosis. Compared to circle fitting and standard Cobb angle measurements, the spline-based approach shows better agreement with clinical gold standards and excellent intra- and inter-rater reliability. By using three key vertebral landmarks the method effectively captures regional spinal curvature, including in cases of severe deformity where traditional methods may falter. These landmarks are well-suited for automated detection by artificial intelligence (AI), enabling fast, consistent and reproducible measurements. The method’s robustness, compatibility with automated workflows and potential correlation with clinical outcomes underscore its clinical relevance for treatment planning, monitoring and surgical decision-making. Future work should validate this approach in larger, more diverse populations and explore its integration into AI-assisted diagnostic systems, focusing on automation.

## Figures and Tables

**Table 1:** Intra-rater reliability of kyphosis angle measurements assessed by ICC(2) for each rater and measurement method. High ICC values close to 1 indicate excellent consistency in repeated measurements by the same rater.

Rater	Method	ICC(2)
A	Spline	0.968
*B*	Spline	0.967
A	Circle	0.973
*B*	Circle	0.970

**Table 2:** Inter-rater reliability of kyphosis angle measurements assessed by ICC(2) for the spline and circle methods. Values close to 1 indicate excellent agreement between raters based on averaged repeated measurements per image.

Method	ICC(2)
Spline	0.979
Spline	0.982

**Table 3:** Pearson correlation coefficients, mean absolute error (MAE) and root mean squared error (RMSE) for the agreement between spline and circle angle measurements and the gold-standard Cobb angles. These metrics assess the linear relationship and average measurement error for each method.

Method	Pearson Correlation Coefficient	*p*	MAE	RMSE
Spline	0.851	<0.001	5.812	8.937
Circle	0.855	<0.001	6.802	9.633

**Table 4:** Receiver operating characteristic area under the curve (ROC AUC), *p*-values from paired *t*-tests and Levene’s tests, Bland–Altman mean bias and limits of agreement (LoA) for each measurement method. These metrics evaluate classification performance, statistical agreement and measurement consistency relative to Cobb angles.

Method	ROC AUC	*t*-Test *p*	Levene’s Test *p*	Bland–Altman Mean	LoA Lower	LoA Upper
Spline	0.96	0.44	0.78	0.930	−16.64	18.51
Circle	0.95	0.06	0.48	2.460	−15.96	20.88

**Table 5:** Sensitivity analysis of kyphosis angle measurements to alternative vertebral landmark triplets for spline and circle fitting methods. Results for the T10–T7–T3 and T12–T9–T5 configurations are presented relative to the standard T12–T8–T4 triplet, which served as the reference. Mean absolute error (MAE), bias, ICC(2,1) and paired *t*-test *p*-values indicate the degree of measurement variation compared to the reference triplet.

Method	Landmark Triplet	MAE (°) ± SD	Bias (°) ± SD	ICC(2,1)	Paired *t*-Test vs. T12–T8–T4 (*p*)
Spline	T10–T7–T3	6.18 ± 4.5	−1.1 ± 8.3	0.964	0.42
Spline	T12–T9–T5	6.47 ± 4.8	−1.5 ± 8.5	0.959	0.35
Circle	T10–T7–T3	7.02 ± 4.7	2.67 ± 9.8	0.961	0.51
Circle	T12–T9–T5	7.28 ± 5.1	2.93 ± 9.9	0.954	0.44

## Data Availability

The data supporting the findings of this study are available from the corresponding author upon reasonable request. To protect participant privacy, the data are not publicly available.
